# Overcoming barriers and enhancing strategies in organ transplantation systems: a systematic review

**DOI:** 10.3389/frtra.2026.1835021

**Published:** 2026-05-22

**Authors:** Priya Sharma, Nipin Kalal, Nimarta Rana

**Affiliations:** College of Nursing, All India Institute of Medical Sciences (AIIMS), Jodhpur, India

**Keywords:** brain death awareness, educational interventions, knowledge-action gap, medical mistrust, organ donation, socio-cultural factors, systematic review, transplantation barriers

## Abstract

**Background:**

Organ transplantation is one of the major achievements of modern medicine, yet the global shortage of organs remains a serious challenge. This shortage increases mortality among patients on waiting lists. Although up to 80% of people support organ donation, far fewer register as donors, creating a clear “knowledge–action gap.”

**Aim:**

This systematic review aimed to identify key barriers to organ donation and evaluate strategies to improve donation rates.

**Methods:**

A systematic search was conducted across PubMed, Scopus, Web of Science, and Google Scholar for peer-reviewed studies published in the last decade. Following PRISMA guidelines, 33 studies were included, covering randomized controlled trials, cross-sectional, and mixed-methods designs. Study quality was assessed using the Joanna Briggs Institute (JBI) Critical Appraisal Tools.

**Results:**

Barriers to organ donation were found to be complex and interconnected, involving social, psychological, and systemic factors. Lack of knowledge was the most common issue, with up to 91% reporting poor awareness and only 11% aware of relevant laws. Sociocultural and religious concerns also influenced decisions, with 38.8% reporting misconceptions and 58% lacking family support. Psychological barriers were significant, with 69% fearing health complications and expressing mistrust in the healthcare system. At the system level, despite general willingness, only 37.3% of healthcare professionals had donor cards. However, targeted interventions showed positive outcomes. Educational programs increased willingness to register from 12% to 68% and improved knowledge from 36.4% to 88.5%.

**Conclusion:**

The main barrier to organ donation is the gap between willingness and action, largely due to uncertainty and lack of understanding. Bridging this gap requires a combined approach: providing simple, culturally appropriate education about donation and improving healthcare systems to build trust and reduce financial and practical burdens on donors.

## 1 Introduction

Organ transplantation is a fundamental part of modern medicine, often the last and most effective treatment for end-stage organ failure. Studies show that successful transplants not only save lives but improve the overall quality of life significantly in patients suffering with chronic diseases (1, 2). However, a chronic worldwide problem still exists that there is shortage of organs in quantity and the need to restore those tissues exceeds supply.

The gap between patients waiting for transplants and available donors continues to widen, leading to high mortality rates for those waiting for a suitable organ (1, 3). Even though there are better surgical techniques and organ allocation rules, the shortage remains the biggest limitation in transplantation (4). Less than less than 10% of the worldwide need for transplantation is actually being met according to current global data (5). This problem is even more acute in low- and middle-income countries, where limited resources, weaker infrastructure and policy limitations further make transplantation more difficult (5–7).

In addition to shortage itself, several systemic “missed opportunities” also contribute to this shortage including delays in identifying potential donors, logistical hurdles in organ retrieval and transport and poor coordination between hospitals (3). Ethical concerns and differences consent processes also makes it complex (8).

Interestingly, the shortage isn't always due to a lack of public support, there are several system-level issues that worsen the situation as there is documented “knowledge-action gap” where people are willing to donate organs, but they never take step to actually register (3). A major reason of this is lack of awareness. Many individuals including university students and the general public do not completely understand the concepts like brain death or who can be a potential donor (2). These informational gaps are widespread and has also been reported in other studies (9, 10).

Beyond this, cultural, religious, and psychological factors also influence the decisions about organ donation. Hesitancy to donate can result due to misconceptions, fear and misinformation regarding the donation process (11). Commonly reported barriers include concerns about bodily integrity and religious interpretations (12–15). In addition, psychological barrier persists due to lack of trust in the health care system particularly the fear of premature declaration of death (15, 16).

Evidence has proved that well-structured educational initiatives and better communication strategies can help increase donation rates (17–19). Also, policy changes that aim to enhance the transparency and simplifying the donation process for families are very crucial (4, 20). It is equally important to consolidate our understanding of them because these barriers or challenges are so complex and interconnected.

This review aims to consolidate the existing literature on the various elements that can play a role in affecting organ transplantation. It focuses on knowledge gaps, socio-cultural factors and addressing systemic constraints (3, 11) along with evaluating the effectiveness of existing interventional strategies in the health care system (4). The ultimate goal is to provide practical, evidence-based recommendations to help bridge the “knowledge–action gap” and improve the outcomes for organ transplantation.

## 2 Materials and methods

### 2.1 Search strategy and data sources

A methodical search was conducted on four primary electronic databases: PubMed, Scopus, Web of Science, and Google Scholar. This search targeted peer-reviewed publications from the last 10 years to ensure that the results are clinically relevant in nature. The search strategy included Boolean operators (AND/OR) with specific keywords such as “organ donation,” “organ transplantation,” “transplantation barriers,” “organ registration,” “brain death knowledge,” and “donation strategies.”

### 2.2 Inclusion and exclusion criteria

Studies were selected based on established inclusion and exclusion criteria to ensure relevance and methodological rigor. The inclusion criteria focused on research studies including randomized controlled trials, cross-sectional studies, and mixed-methods studies specifically focused on barriers, attitudes, perceptions, or strategies for organ donation and transplantation. Systematic reviews, editorials, commentaries, and conference abstracts; studies which were not associated with organ donation or transplantation frameworks, studies which were lacking adequate data on barriers or strategies, not published in English language; and studies where full texts could not be accessed were excluded.

### 2.3 Data extraction and quality assessment

A total of 1,139 records were identified initially, after which 139 duplicates were removed. The remaining 1,000 studies were then screened by two independent reviewers using Rayyan software, resulting in selection of 129 abstracts for additional review. Of these, 59 articles were evaluated for full-text eligibility, and finally 33 studies met the inclusion criteria and were included in the analysis. Data extraction was completed using a standard form that included details such as author, year, geographic setting, study design, sample size, and the main findings. To be ensured about the reliability of the evidence, the quality of the included studies was assessed using the Joanna Briggs Institute (JBI) Critical Appraisal Tools. Most studies were evaluated using the JBI checklist for cross-sectional studies, while interventional studies were assessed using the checklist for randomized controlled trials (see Table 1). Each study was then independently assessed for potential bias in the sampling, methods, measurement techniques used, and statistical analysis. Studies who met the high-quality standards were included to ensure a reliable synthesis of data.

**Table 1:** JBI-based quality assessment of included studies.

Author (Year)	Study Design	Appraisal Tool (JBI-based)	Score (8)	Quality Level
Kaplow et al. (26)	Survey	Adapted JBI (Cross-sectional domains)	8	High
Bas-Sarmiento et al. (31)	RCT	Adapted JBI (RCT domains)	8	High
Al-Marzouqi et al. (35)	Cross-sectional	Adapted JBI (Cross-sectional domains)	7	High
Thornton et al. (36)	RCT	Adapted JBI (RCT domains)	8	High
Moskal-Szybka & Borek (21)	Survey	Adapted JBI (Cross-sectional domains)	6	Moderate
Alshehri et al. (9)	Cross-sectional	Adapted JBI (Cross-sectional domains)	8	High
Al-Salhi & Othman (10)	Cross-sectional	Adapted JBI (Cross-sectional domains)	8	High
Hasan et al. (12)	Cross-sectional	Adapted JBI (Cross-sectional domains)	7	High
Abdulrazeq et al. (22)	Mixed-methods	Adapted JBI (Mixed domains)	7	Moderate
Quick et al. (27)	RCT	Adapted JBI (RCT domains)	8	High
Patthi et al. (19)	Quasi-experimental	Adapted JBI (Quasi-experimental domains)	7	Moderate
Smith et al. (28)	Mixed-methods	Adapted JBI (Mixed domains)	8	High
Siminoff et al. (29)	RCT	Adapted JBI (RCT domains)	8	High
Khoshravesh et al. (2021)	RCT	Adapted JBI (RCT domains)	8	High
Arizmendi-Villarreal et al. (33)	Cross-sectional	Adapted JBI (Cross-sectional domains)	7	Moderate
Alshamsi et al. (37)	Cross-sectional	Adapted JBI (Cross-sectional domains)	8	High
Asanova et al. (17)	RCT	Adapted JBI (RCT domains)	8	High
Hvidt et al. (25)	Mixed-methods	Adapted JBI (Mixed domains)	7	Moderate
Oczkowski et al. (30)	Cross-sectional	Adapted JBI (Cross-sectional domains)	8	High
Alaamri et al. (23)	Cross-sectional	Adapted JBI (Cross-sectional domains)	7	Moderate
Bertocchi et al. (38)	Cross-sectional	Adapted JBI (Cross-sectional domains)	8	High
Khaleq et al. (13)	Cross-sectional	Adapted JBI (Cross-sectional domains)	6	Moderate
Sree Harichandana et al. (18)	Quasi-experimental	Adapted JBI (Quasi-experimental domains)	8	High
Ali et al. (39)	Cross-sectional	Adapted JBI (Cross-sectional domains)	7	Moderate
Krupic Ferid (32)	Qualitative/intervention	Adapted JBI (Qualitative domains)	8	High
Bolatov et al. (15)	Cross-sectional	Adapted JBI (Cross-sectional domains)	8	High
Tarzi et al. (14)	Cross-sectional	Adapted JBI (Cross-sectional domains)	7	Moderate
Boadu et al. (40)	Mixed-methods	Adapted JBI (Mixed domains)	8	High
Hanifa et al. (41)	Cross-sectional	Adapted JBI (Cross-sectional domains)	7	Moderate
Oo et al. (34)	Cross-sectional	Adapted JBI (Cross-sectional domains)	8	High
Akbulut et al. (42)	Cross-sectional	Adapted JBI (Cross-sectional domains)	7	Moderate
Pekmezaris et al. (16)	RCT	Adapted JBI (RCT domains)	8	High
Al-Sharbatti et al. (24)	Cross-sectional	Adapted JBI (Cross-sectional domains)	8	High

### 2.4 Data synthesis and thematic analysis

A thematic analysis approach was employed to examine the results across the 33 included studies. Data was categorized into four main areas of resistance: knowledge deficits, socio-cultural and religious ambiguity, psychological obstacles, and systemic or financial limitations. Intervention strategies were also classified on the basis of their focus areas, such as specific educational programs, emotional or culturally tailored communication models, and policy reforms aimed at financial neutrality. This synthesis facilitated a thorough mapping of the “knowledge-action gap” identified within the existing literature.

## 3 Results

### 3.1 Study selection

**Figure 1:**
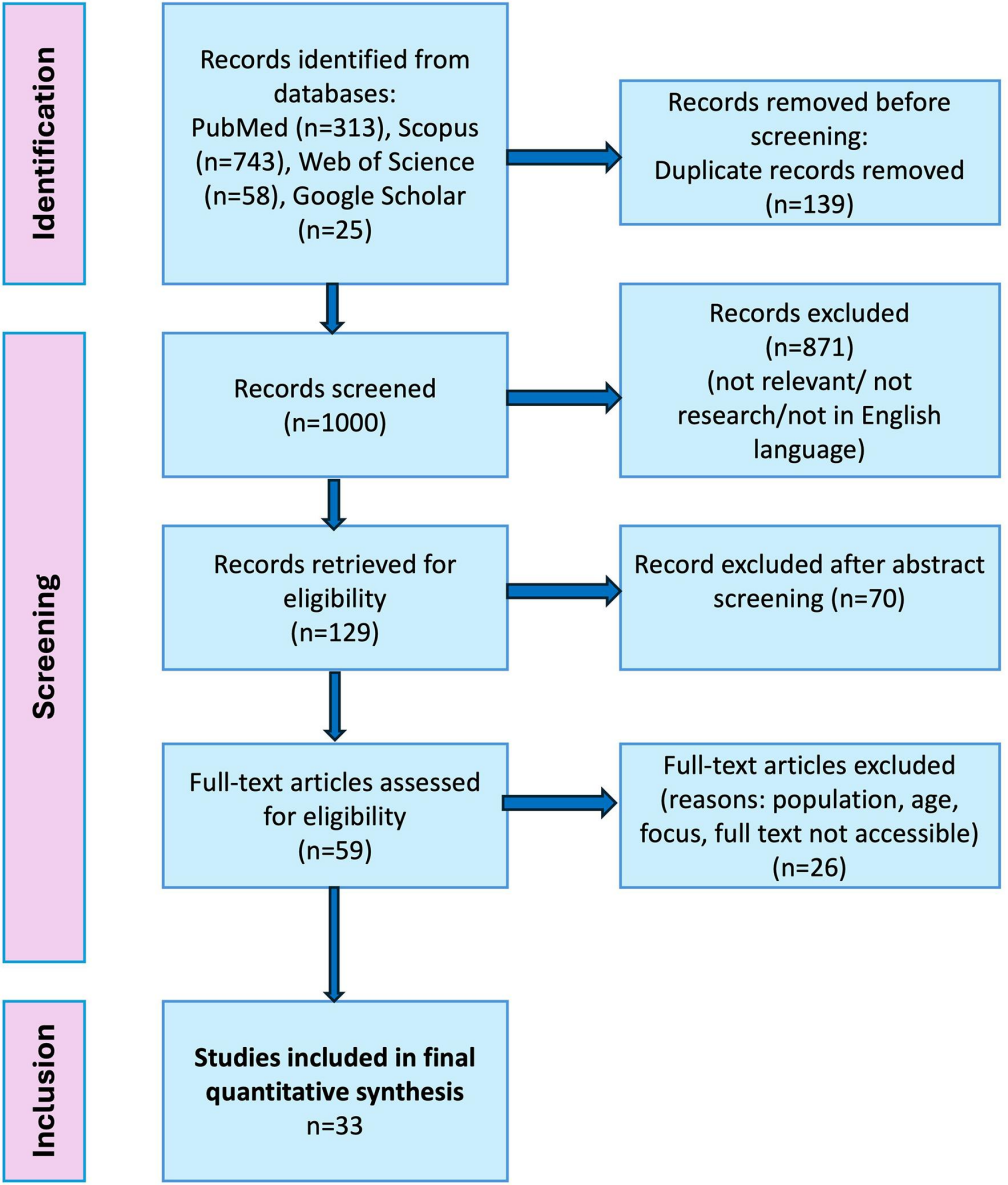
PRISMA flow diagram illustrating the study selection process.

The search strategy resulted in an initial count of 1,139 records from four databases: PubMed (*n* = 313), Scopus (*n* = 743), Web of Science (*n* = 58), and Google Scholar (*n* = 25). After the removal of 139 duplicates, 1,000 unique studies retained for title screening. During this screening phase, 871 records were excluded that did not meet our inclusion criteria, because of being off-topic, not an original research, or published in a languages other than English. This reduced the number to 129 abstracts, of which 70 were subsequently removed because they were lacking relevance to our primary research objectives. Of the remaining 59 articles that were evaluated for full-text eligibility, 26 studies were excluded due to incompatible study populations, a lack of focus on specific barriers or strategies, publication dates exceeding our 10-year threshold, or the unavailability of full text. Aa a result, 33 studies fulfilled the inclusion criteria and were selected for final quantitative analysis. The study selection process of the selected studies has been illustrated in Figure 1.

### 3.2 Study characteristics

To provide a detailed overview of the current literature, we categorized the 33 selected studies based on the design, population demographics, and geographic locations (see Table 2). The studies used different methods, including randomized controlled trials, mixed-methods research, and cross-sectional surveys. This helped us to see how organ donation is perceived across different groups including clinicians, university students, patients, and general public from various parts of world.

**Table 2:** Characteristics of included studies (*N* = 33).

Author (Year)	Title	Study Design	Setting & Population	Key Findings & Barriers Identified
Kaplow et al. (26)	National Attitudes Toward Living Kidney Donation in the United States: Results of a Public Opinion Survey	National Public Opinion Survey	802 US adults (aged 25–65)	**Barriers:** Surgical risks, medical expenses/financial disincentives, and potential long-term health effects. **Facilitators:** Education on surgical safety, removal of financial costs, and awareness of kidney-paired donation.
Bas-Sarmiento et al. (31)	Intervention programme to improve knowledge, attitudes, and behaviour of nursing students towards organ donation.	Randomised Controlled Trial (RCT)	73 s-year nursing students at the University of Cadiz, Spain	**Barriers:** Lack of knowledge, negative attitudes, and lack of clinical experience. **Results:** Educational interventions significantly improved knowledge, attitudes, and willingness to discuss donation with families.
Al-Marzouqi et al. (35)	Awareness, attitudes and barriers to organs donation in Oman: A cross sectional study	Cross-sectional study	622 residents of North Batinah Governorate, Oman	**Barriers:** Fear of health complications from living donation (69%), family objection (38%), and lack of knowledge (91%). **Key Finding:** While 58% had positive attitudes, cultural/religious norms and family consensus heavily influence decisions.
Thornton et al. (36)	Effects of a Video on Organ Donation Consent Among Primary Care Patients: A Randomized Controlled Trial	Randomized Controlled Trial (RCT)	915 patients in 18 primary care clinics in Cuyahoga County, Ohio, USA	**Barriers:** Lack of information to allay fears/concerns and infrequent physician-patient discussion due to time or training. **Key Finding:**Brief video interventions and physician cueing increased consent rates from 15% to 22%.
Moskal-Szybka & Borek (21)	Knowledge and attitudes of Subcarpathian Province's inhabitants towards organ transplantation	Diagnostic survey (Questionnaire)	187 non-medical inhabitants of Subcarpathian Voivodeship, Poland	**Barriers:** Moderate to low public awareness, fear of donation, and lack of knowledge about the concept of brain death. **Key Finding:** Most respondents expressed willingness to donate, but identified a strong need for public education to achieve full acceptance.
Alshehri et al. (9)	Knowledge and willingness to donate kidney for transplantation among general population in Saudi Arabia	Cross-sectional study	1,012 members of the general population in the Aseer region, Saudi Arabia	**Barriers:** Fear of health complications (43.5%), lack of information/awareness, and religious concerns. **Key Finding:** While knowledge levels were moderate, a significant majority (80.1%) expressed willingness to donate to a relative, but only 34.6% to a stranger.
Al-Salhi & Othman (10)	Public knowledge about and attitudes toward organ donation, and public barriers to donate in Jordan	Cross-sectional study	572 residents across various regions of Jordan	**Barriers:** Lack of knowledge (89%), lack of family support (57%), and distrust in the medical system/fear of organ trade (54.5%). **Key Finding:** Knowledge was significantly low; educational levels and previous exposure to organ donation information were predictors of positive attitudes.
Hasan et al. (12)	Insight into the Knowledge, Attitude, Practices, and Barriers Concerning Organ Donation Amongst Undergraduate Students of Pakistan	Cross-sectional study	450 medical and non-medical undergraduate students in Karachi, Pakistan	**Barriers:** Religious misconceptions (38.8%), lack of transparency in the donation process, and fear of surgical risks. **Key Finding:** Medical students had higher knowledge than non-medical students, yet 62.1% of the total sample felt there was a need for more awareness campaigns.
Abdulrazeq et al. (22)	Barriers in Knowledge and Attitudes Regarding Organ Donation among Urban Jordanian Population	Mixed-methods (Qualitative interviews & Quantitative survey)	396 residents of Amman, Jordan	**Barriers:** Lack of knowledge regarding “brain death,” religious ambiguity (42.2% unsure of Islamic stance), and bodily integrity concerns. **Key Finding:** High support for donation in theory, but low actual registration due to lack of procedural knowledge.
Quick et al. (27)	Can Happiness and Sadness Overcome Organ Donation Barriers Following Exposure to Radio Ads?	Randomized Controlled Trial (RCT)	652 US adults (via Amazon Mechanical Turk)	**Barriers:** “The Ick” (disgust/bodily integrity), “Medical Mistrust” (fear of premature declaration of death), and “Self-Interest.” **Key Finding:** Radio ads evoking sadness or happiness effectively reduced barriers compared to a control, with sadness particularly lowering “The Ick” and medical mistrust.
Patthi et al. (19)	Beliefs and barriers for organ donation and influence of educational intervention on dental students: A questionnaire study	Pre- and post-intervention study	453 dental students in Modinagar, India	**Barriers:** Lack of awareness (initially 36.4%), religious beliefs, and fear of misuse of organs. **Key Finding:** Educational interventions significantly improved knowledge (from 36.4% to 88.5%) and positive attitudes toward donation.
Smith et al. (28)	Living Donor Decision-Making and the Complex Interplay of Finances and Other Motivators, Barriers, and Facilitators	Mixed-methods study	114 living donors and 13 individuals who declined donation (USA)	**Barriers:** Lost wages, travel costs, and concerns about long-term health/insurance. **Key Finding:** Financial burdens are significant but often secondary to the emotional relationship with the recipient; however, financial “neutrality” is essential for equitable access.
Siminoff et al. (29)	Communicating Effectively About Organ Donation: A Randomized Trial.	Randomized Controlled Trial (RCT)	1,603 family decision-makers and 273 requesters (USA)	**Barriers:** Poor communication quality, lack of rapport, and inadequate explanation of brain death. **Key Finding:** Advanced communication training (CEaD) for hospital staff significantly increased family authorization rates for organ donation.
Khoshravesh et al. (2021)	Evaluation of a workplace organ donation intervention: A randomized controlled trial	Randomized Controlled Trial (RCT)	Employees in Hamadan, Iran	**Barriers:** Lack of information regarding the donation process and misconceptions about the legal/religious framework. **Key Finding:** Targeted workplace education significantly increased the registration of donor cards.
Arizmendi-Villarreal et al. (33)	Strategic insights into organ donation: perceptions, attitudes, and the impact of disincentive removal in current and future medical professionals	Cross-sectional study	Medical professionals and students in Mexico	**Barriers:** Fear of medical negligence, distrust in transplantation systems, and financial disincentives. **Key Finding:** Removing financial barriers (e.g., costs associated with donation) and professional education is essential to improve donation rates.
Alshamsi et al. (37)	Attitudes toward organ donation among university students in the United Arab Emirates, a cross-sectional survey	Cross-sectional study	University students in the UAE	**Barriers:** Cultural and religious ambiguity, and insufficient knowledge about the technical aspects of transplantation. **Key Finding:** Despite positive attitudes, lack of formal awareness programs acts as a barrier to actual registration.
Asanova et al. (17)	From Uncertainty to Consent: Educational Intervention Effects on Knowledge and Willingness to Donate Organs After Death	Randomized Controlled Trial (RCT)	General population (targeted for intervention)	**Barriers:** High levels of “uncertainty” stemming from inadequate knowledge about brain death and the consent process. **Key Finding:** Structured educational interventions successfully transformed “uncertainty” into “willingness to consent.”
Hvidt et al. (25)	For and against Organ Donation and Transplantation: Intricate Facilitators and Barriers in Organ Donation Perceived by German Nurses and Doctors	Mixed-methods study	179 nurses and doctors in Germany	Barriers: Physical body integrity concerns (“that ODT violates the body”) and personal discomfort in proposing donation to grieving relatives. **Key Finding:** Religious arguments are increasingly used to favor donation rather than oppose it; however, barriers differ between healthcare professionals' personal views and their perceptions of what relatives believe.
Oczkowski et al. (30)	A Multidisciplinary Survey to Assess Facilitators and Barriers to Successful Organ Donation in the Intensive Care Unit	Cross-sectional survey	108 ICU staff (nurses, respiratory therapists, physicians) in an academic tertiary care hospital	**Barriers:** Challenges in team communication, ambiguity regarding roles throughout the donation request process, and perceived delays in clinical support for families. **Key Finding:** Effective collaboration within a multidisciplinary team and prompt communication are essential components that facilitate successful donation workflows in the ICU.
Alaamri et al. (23)	Knowledge, Attitudes, and Barriers of Organ Donation in Jeddah City, Saudi Arabia: A Cross-sectional Study	Cross-sectional study	600 + residents of Jeddah, Saudi Arabia	**Barriers**: A significant portion of individuals report apprehension towards the “unknown” (31.2%), a lack of understanding about the donation process (28.5%), and a general unawareness of both local and international regulations (73%). **Key Finding:** Although half of the population expresses a willingness to donate, access to pertinent information predominantly depends on online resources rather than formal educational avenues, perpetuating enduring misconceptions.
Bertocchi et al. (38)	Knowledge and Attitudes Toward Organ Donation and Transplantation Among Nursing Students: A Multicentre Cross-Sectional Study	Multicentre Cross-sectional study	Nursing students in Italy	**Barriers:** Insufficient formal curriculum coverage, ethical dilemmas regarding the definition of death, and emotional impact of the donation process.
Khaleq et al. (13)	Organ Donation and Obstacles: University Student Survey	Cross-sectional survey	240 university students in Morocco	**Barriers:** Strong religious hesitation (perceived conflict with Islamic tenets), lack of trust in national transplant systems, and fear of mutilation.
Sree Harichandana et al. (18)	An Interventional Health Education Study to Transition the General Population's Opinion on Organ Donation From Reluctance to Acceptance	Pre-post intervention study	General population in Chennai, India	**Barriers:** Misconceptions related to body disfigurement, insufficient awareness, and familial impact. **Finding:** A well-organized educational program notably enhanced the readiness to obtain a donor card, rising from 12% to 68%.
Ali et al. (39)	Knowledge and Attitude of People With or Without a Medical Education Regarding Organ Donation and Transplant: A Sample From the City of Baghdad	Cross-sectional study	400 residents of Baghdad, Iraq	**Barriers:** Deep mistrust of systemic transparency, cultural misconceptions, and lack of awareness of local legislation (only 11% knew the law).
Krupic Ferid (32)	The impact of religion and provision of information on increasing knowledge and changing attitudes to organ donation: an intervention study.	Intervention/Focus group study	36 religious immigrants in Sweden	**Barriers:** Lack of information tailored to specific religious needs and fear of the “unknown.” **Finding:** Culturally sensitive information effectively bridges the gap between religious practice and medical donation needs.
Bolatov et al. (15)	Barriers and willingness to express consent to organ donation among the Kazakhstani population	Cross-sectional survey	1,294 adults aged ≥18 years from various regions of Kazakhstan recruited through online platforms	**Barriers:** Distrust in the healthcare system, apprehensions about organ trafficking, cultural and religious beliefs, and insufficient comprehension of organ donation. **Key Findings:** Factors such as awareness, age, ethnicity, marital status, and education level significantly impact individuals' willingness to donate organs. Those who possess greater knowledge about organ donation are more inclined to consent to it, underscoring the necessity for focused educational initiatives aimed at enhancing donation rates.
Tarzi et al. (14)	Attitudes towards organ donation in Syria: a cross-sectional study	Cross-sectional survey	303 participants recruited from four hospitals in Aleppo, Syria	**Barriers:** Concerns about bodily disfigurement post-mortem, insufficient understanding of brain death, and a lack of familiarity with organ donation regulations and policies. **Key Findings:** While 58% of participants were in favor of organ donation and 62% indicated a willingness to donate their organs, a small number possessed sufficient knowledge regarding brain death and the associated legal frameworks. It was suggested that educational initiatives and awareness programs related to policies should be implemented to encourage organ donation after death.
Boadu et al. (40)	Subgroup differences in public attitudes, preferences and self-reported behaviour related to deceased organ donation before and after the introduction of the “soft” opt-out consent system in England: Mixed-methods study	Mixed-methods study (cross-sectional surveys + qualitative interviews)	National datasets including 19,011 survey participants and 30 qualitative interview participants from England	Barriers: Lack of awareness regarding the opt-out policy, cultural and religious influences, misinformation, and difficulties discussing organ donation decisions with family members. Key Findings: Public endorsement for organ donation consistently stood at approximately 80%, although it was notably lower within minority ethnic communities. The research delineated various categories of donor attitudes and emphasized the critical role of conversations among family members, focused public outreach initiatives, and culturally attuned strategies to enhance consent rates.
Hanifa et al. (41)	Attitudes of healthcare students in Syria toward organ donation and their association with healthcare system distrust	Cross-sectional survey	615 healthcare students from medicine, dentistry, and pharmacy faculties at the University of Kalamoon, Syria	**Barriers:** Skepticism regarding the healthcare system, worries about privacy and the potential exploitation of organs, as well as cultural and religious considerations, contribute to hesitance in discussing organ donation with relatives. **Key Findings:** Generally, students exhibited a moderately favorable perspective on organ donation. But, there remains a significant level of distrust towards the healthcare system.
Oo et al. (34)	Knowledge and attitudes of healthcare professionals and the impact on willingness to donate organs: A tertiary hospital survey	Cross-sectional survey	412 healthcare professionals working in critical care units at Hospital Kuala Lumpur, Malaysia	Barriers: Insufficient understanding of brain death, cultural and religious beliefs, low trust in transplantation procedures, and inadequate awareness regarding organ donation processes. Key Findings: Approximately 68% of healthcare professionals expressed a willingness to donate organs; however, only 37.3% possessed donor cards, while 63.1% had discussed their intentions with family members. Factors such as awareness regarding brain death, religious beliefs, and professional roles influenced their willingness to donate. This study suggests targeted education for healthcare professionals to improve donation rates.
Akbulut et al. (42)	Attitudes, awareness, and knowledge levels of the Turkish adult population toward organ donation: Study of a nationwide survey	Nationwide cross-sectional survey	3,000 adults aged ≥18 years from 26 regions across Turkey	**Barriers**: Limited knowledge regarding organ donation and the concept of “brain death,” apprehensions concerning the integrity of the body after death, religious beliefs, and concerns about the premature retrieval of organs. **Key Findings**: While the majority of participants expressed their willingness to undergo organ transplantation if needed, very few had considered donating their own organs. A lack of understanding regarding organ donation was observed among the general public, underscoring the need for comprehensive educational initiatives and policy measures aimed at increasing organ donation rates.
Pekmezaris et al. (16)	Randomized intervention to assess the effectiveness of an educational video on organ donation intent among Hispanics in the New York metropolitan area	Randomized intervention study	365 Hispanic adults (≥18 years) residing in the New York metropolitan area, United States	Barriers: Medical mistrust, fear related to bodily integrity, discomfort with organ donation (“ick” feelings), and superstition that registering as a donor may hasten death (“jinx” belief). Key Findings: Participants exposed to an emotive educational video before completing the survey were significantly more likely to express willingness to register as organ donors. Targeted educational messaging was effective in improving donation intentions among minority populations.
Al-Sharbatti et al. (24)	Attitudes, and barriers towards organ donation among university students, faculty and staff in Ajman, United Arab Emirates: Cross-sectional survey design	Cross-sectional survey	607 university students, faculty, and staff from three universities in Ajman, UAE	Barriers: Lack of awareness about organ donation (64.1%), fear related to medical procedures (51.9%), concerns regarding misuse of organs, and uncertainty about religious support. Key Findings: Although many participants supported organ donation conceptually, uncertainty regarding actual registration remained high. Supportive attitudes were significantly associated with knowledge level, demographic factors, and previous exposure to organ donation. Educational programs were recommended to improve awareness and promote positive attitudes.

The barriers to organ donation and not simple or isolated instead they are interconnected and are influenced by many factors. These barriers can be categorized into four categories such as lack of clear information, uncertainty regarding cultural or religious beliefs, fears about the process of organ donation, and gaps in healthcare system. Upon looking at these factors together, we can better identify the barriers and more importantly, can rule out the best ways to address them.

### 3.3 Barriers influencing organ donation

Our synthesis of the 33 included studies identified the barriers to organ donation are not isolated but interconnected. Instead, these challenges arise from a complex web of social, psychological, and systemic related factors. Across different population, there were four main types that were identified.

#### 3.3.1 Knowledge gaps

The most commonly reported barrier is not a lack of willingness to help, but a lack of proper knowledge. Many people shown willingness to donate organ in theory but hesitate because of lack of understanding about important concepts like brain death, the legal process of consent, and the steps involved in transplantation. Studies conducted across multiple regions reported insufficient awareness and understanding of organ donation procedures among the general population and healthcare professionals (9, 10, 21–23)^.^ This confusion often prevents well-intentioned individuals from taking the final step to register as donors (9, 24).

#### 3.3.2 Cultural and religious ambiguity

Cultural and religious perspectives play a significant role in influencing decisions about organ donation. Several studies revealed that people are unsure regarding their religious acceptance of organ donation creating hesitation among potential donors (12, 13). In many communities, beliefs about keeping bodily integrity after death also plays a role, which can create a moral or cultural conflict for individuals considering organ donation (14, 15). Research among healthcare professionals and the general population also suggests that individual's personal interpretations of the religious teachings and cultural norms can also influence willingness to donate significantly (25).

#### 3.3.3 Psychological perception and fear

Fear is also an important barrier to organ donation and is reported across different populations. Concerns include fear of surgical complications in living donation, mistrust of the healthcare system, and anxiety related to illegal activities like trafficking (15, 26). Psychological discomfort related to the idea of organ removal after death has also been identified as a factor (16). Emotional reactions such as fear, distrust or discomfort toward organ removal have been identified as factors influencing willingness for organ donation (27).

#### 3.3.4 Systemic and financial realities

Apart from personal beliefs and fears, structural and financial challenges affects organ donation decisions. Potential living donors frequently report concerns such as indirect costs associated with donation, including lost wages, travel expenses, and the post operative recovery period (26, 28). Studies assessing healthcare system practices highlights organizational barriers, such as inadequate communication between healthcare professionals and the families during the process of organ donation (29, 30). These factors can affect family authorization decisions and ultimately limit successful organ donation.

### 3.4 Strategies to improve organ donation

The literature review identifies that the barriers to organ donation are not insolvable; rather, they can be effectively managed through targeted, evidence-based strategies. This analysis also suggests that the most successful approaches are those that directly address the gaps in knowledge, psychological fears related to donation, and structural inadequacies that were identified earlier in the previous section.

#### 3.4.1 Targeted educational interventions

Education is a vital tool through which public attitudes towards organ donation can be shifted from opposition to acceptance. Research consistently demonstrates that well-structured programs can significantly narrow the knowledge gap. Studies (19, 31) found that targeted training for nursing and dental students led them to improve their knowledge base and their willingness to discuss donation with families. Similarly, for the general public, interventions have been shown to be effective in transforming high levels of “uncertainty” into formal consent by providing clear information regarding brain death and the legal registration process (17, 18).

#### 3.4.2 Strategic communication and media

The most-needed are good communication strategy; one that goes beyond the simple reporting of facts, in order to connect with people at an emotional/cultural level in donation choices. Results show the messages are as important as what they convey. One study (27) found, for example, that radio programmes that provoked particular emotions such as sadness or joy were more effective in reducing the feelings of “medical mistrust” and “disgust” than standard information campaigns. Additionally studies with immigrant groups emphasize the value of culturally-based video messages in correcting misconceptions of fears of death hastened by organ donation (16, 32).

#### 3.4.3 Institutional and policy reform

The literature points out individual willingness is often met with obstacles in healthcare systems, therefore institutional reforms are critical in enabling that willingness. One study (28) illustrated that the CEaD model-specific advanced communication training significantly increased family authorization rates through facilitating the establishment of rapport between hospital staff and clinicians, as well as through clearer clinical instruction during donation discussions. In addition, policy measures, such as dismantling financial disincentives towards living donors, represent the first steps towards achieving the goal of “financial neutrality” and also to developing a more equitable donor setting overall (1, 28, 33). When healthcare institutions put such reforms first, they actively facilitate rather than impede the decision to donate organs.

## 4 Discussion

### 4.1 Principal findings and the knowledge-action gap

The review of 33 included studies identified that the principal barrier to organ donation is persistent “knowledge–action” gap. Whilst there is considerable public support for organ donation—with approval levels approaching 80%—particularly in England, reporting that a substantial disconnect persists when individuals are required to translate this willingness into formal registration. This hesitation stems from a lack of formal knowledge regarding the medical definition of “brain death,” legal consent procedures, and registration processes. Studies (21–23) have consistently reported that this uncertainty is a global phenomenon, observed across various regions. This pattern of low awareness has been identified among the general public in Saudi Arabia and Jordan (9, 10), as well as among healthcare professionals, who have demonstrated gaps in their knowledge regarding donation protocols (33). Therefore, the findings suggest that hesitation may be driven by uncertainty rather than outright opposition; furthermore, the observed detachment appears to result from a lack of procedural understanding rather than a lack of altruistic intent. Ultimately, the most effective means of shifting public attitudes involves systematic educational interventions that provide clear and practical information regarding the transplantation process (17, 18).

### 4.2 Socio-cultural and religious determinants

Beyond mere ignorance of this nature, there exist complex webs woven from socio-cultural beliefs ranging from religious ambiguity to outright opposition to accepting any form of post-mortem organ donation. Numerous studies indicate that the uncertainty directly stemming from concerns regarding religious sanction is a major cause of delays in decision-making; Muslim-majority regions are particularly affected by this (12, 13). Furthermore, shared fears regarding the preservation of bodily integrity after death have reinforced the hesitation observed in recent initiatives undertaken in Syria and Kazakhstan (14, 15). However, there is growing evidence suggesting that religion does not inherently act as a barrier; rather, misinterpretation or a lack of clear guidance can give rise to uncertainty. For instance, research conducted among healthcare professionals (25) demonstrates that religious arguments can also be utilized in a constructive manner to advocate for organ donation. To overcome these barriers, the evidence suggests that a shift toward context-specific messaging represents the right path forward. Interestingly, emotional communication strategies—such as radio or video campaigns—have proven more successful than the mere dissemination of standard information in mitigating “medical mistrust” and feelings of aversion (16, 27, 43).

### 4.3 Psychological barriers and medical mistrust

Fear and systemic mistrust creates profound psychological deterrents that require institutional reform to overcome. People worry about the concerns regarding surgical risks and long-term health consequences especially regarding living donation (1, 28). Dissatisfaction with healthcare systems was another prominent issue, with participants expressing fears of organ misuse, trafficking, or premature declaration of death (15, 16). These affective responses, including the “ick” factor (27) indicate that donation decisions are shaped by perceptual responses that require different intervention strategies. This analysis emphasize that success is dependent on the quality of clinical interaction; specialized training (29). On the other hand, CEaD model, enables healthcare staff to establish necessary rapport with grieving families to improve authorization rates. Furthermore, authorization can also get affected even when potential donors are identified due to organizational challenges and communication gaps (30).

### 4.4 Structural barriers and policy-level interventions

Ultimately, system-level barriers and financial realities play a significant role in limiting donation. Financial concerns such as lost wages, travel expenses, and recovery costs—consistently emerge among potential living donors (1, 28). Policy measures designed to achieve “financial neutralization” are essential for creating an equitable environment for donation (32). These findings suggest that a dual strategy is required to increase organ donation rates one that focuses on both individual perceptions and structural deficiencies. Educational efforts should be centered on targeted training, particularly for healthcare students (19, 31, 34). By combining emotionally and culturally relevant messaging with institutional reforms, financial and psychological risks can be mitigated or managed; in this way, the gap between theoretical willingness and life-saving action can be effectively bridged.

### 4.5 Strengths

The strongest aspect of this systematic review is its evidence base, comprising 33 studies drawn from various countries and the internet; these were sourced from databases such as PubMed, Scopus, and Web of Science. Adhering to PRISMA-guided selection processes, this analysis presents a comprehensive overview of both barriers and evidence-based interventions (e.g., the Siminoff et al. communication model) (29). Furthermore, the diversity in demographics ranging from healthcare providers to the general public ensures that the key themes identified herein namely, the “knowledge-action gap” and “medical mistrust” remain relevant within a broad global perspective.

### 4.6 Limitations

Due to the minimum publication timeframe of 10 years and the restriction to the English language, relevant long-term literature or regional data published in other languages may have been overlooked. Furthermore, given the significant variability in the designs of the 33 included studies, it is possible that some of these findings may not be applicable to all clinical settings. Inclusion of healthcare workers may have introduced sample heterogeneity, limiting generalizability as organ donation barriers vary across regions and religions. Finally, since many studies utilized self-report surveys, a risk of social desirability bias exists in those studies a concern underscored by claims that participants' expressed willingness to donate does not necessarily correlate directly with their actual registration or consent to donate.

## 5 Conclusion

This review of 33 international studies demonstrates the root cause of low organ donation is not so much from a lack of kindness as it is a “knowledge-action gap.” But despite the fact that nearly everyone tends to say that they support donation in principle with support as high as 80 percent in some countries few people sign up to be donors at all. This is so as people typically feel a lack of understanding of what brain death is or how a legal registration process works. To alleviate this, we must shift away from generic advertisements and instead utilize pedagogical approaches that communicate with individuals' feelings and societies. Healthcare workers must also be trained in more effective communication, since patients can gain more consent through open and honest conversations with families, researchers show. Lastly, the system must help living donors financially by preventing them from losing money on travel or absent work. So, saving more lives through organ transplant requires two important actions—making it easier for the public to appreciate the process and reforming the hospitals themselves that organ donations are handled through. It will be finally when the public is well informed and the medical system assists that we will be on the path to closing the gap between “wanting to help” and “taking action”.

## Data Availability

The data analyzed in this study is subject to the following licenses/restrictions: Data will be available with request. Requests to access these datasets should be directed to Nipin Kalal, Kalalnipin@gmail.com.
